# Lactose malabsorption and intolerance in older adults

**DOI:** 10.1097/MCO.0000000000001045

**Published:** 2024-06-06

**Authors:** Antonella Gallo, Emanuele Marzetti, Simona Pellegrino, Massimo Montalto

**Affiliations:** a Fondazione Policlinico Universitario “A. Gemelli” IRCCS; b Department of Geriatrics, Orthopedics and Rheumatology, Università Cattolica del Sacro Cuore, Rome, Italy

**Keywords:** frailty, intolerance, lactose, malabsorption, nutrition, older

## Abstract

**Purpose of review:**

Lactose malabsorption and intolerance are very common conditions. However, their optimal approach, including the diagnostic assessment, remains a matter of debate, especially in advanced age. In this brief review, we focused on current knowledge, concerns, and impact in clinical practice of lactose malabsorption and intolerance in elderly.

**Recent findings:**

Older adults are at high risk of malnutrition, owing to frequent occurrence of cognitive impairment, loss of appetite, dysphagia, and poor oral health. A significant decrease in the consumption of dairy products may lead to inadequate intake of high-quality protein and minerals, with a consequent impact on muscle and bone health. Testing for lactose malabsorption may be challenging in older adults, if not useless. Instead, a detailed clinical evaluation should always be pursued to identify both lactose intolerance and all confounding factors mimicking the same clinical picture.

**Summary:**

The management of lactose malabsorption and intolerance in older adults deserves a personalized approach. Because of the importance of maintaining an adequate nutritional status in this age group, efforts should be put forth to avoid excessively restrictive diets.

## INTRODUCTION

Lactose malabsorption and intolerance are very common conditions, involving all age groups [1]. Although the first entity clearly represents a pathophysiological process, the second one is a clinical picture, the two terms are often used interchangeably, leading to misunderstanding among patients and clinicians [1].

Lactose malabsorption and intolerance have attracted significant research interest over the years. However, uncertainties concerning diagnostic tests, clinical management, and nutritional implications of restrictive diets are still a matter of debate, mainly in particular patient categories, such as older adults.

In this brief review, we focused on current knowledge, existing concerns, and impact on health status of lactose malabsorption and intolerance in older adults. 

**Box 1:**
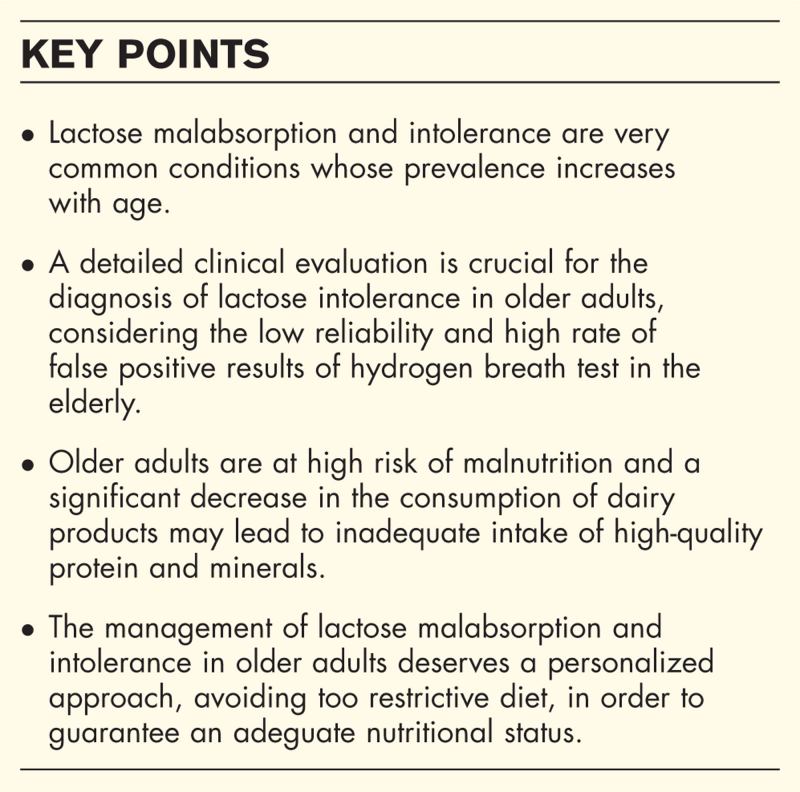
no caption available

## DIET AND AGING

The number of individuals older than 65 years is continuously growing worldwide, which requires to increase awareness on the complexity of the aging process, in order to improve understanding on main age-related disease mechanisms and support research for promoting healthy aging. Robust evidence indicates that modifiable risk factors, including physical activity and nutrition, can play a pivotal role in these contexts [2^▪▪^,^3^^▪▪^,^4^,^5^^▪▪^,6^▪^,^7^].

Regarding nutrition, studies have shown that different dietary components, such as carbohydrates, protein, and lipids, are instrumental in modulating the aging processes and the development of major chronic degenerative diseases, such as cardiovascular and cerebrovascular disease, sarcopenia, and dementia [2^▪▪^,^3^^▪▪^,^4^,^5^^▪▪^,6^▪^,^7^–^9^,10^▪^,^11^]. Despite this body of evidence, older adults remain at high risk of malnutrition [12] because of cognitive impairment, loss of appetite, dysphagia, and poor oral health that contribute to reduce intake of adequate amounts of nutrients [12], mainly in those living in residential care facilities [10^▪^]. The health benefits conveyed by well balanced diet, such as the Mediterranean diet, are indisputable [6^▪^]. Indeed, the synergistic action of different macronutrients and micronutrients, rather than individual dietary agents, may have a significant impact on maintaining muscle mass, strength, and function in age [5^▪▪^]. In this context, milk and dairy products are an important source of nutrients such as protein, calcium, magnesium, potassium, selenium, zinc, vitamin A, vitamin B12, riboflavin, and pantothenic acid [13,14^▪▪^]. Moreover, dairy products are rich in leucine, contributing to muscle anabolism and bone maintenance [11]. An adequate calcium intake may prevent bone loss and osteoporotic fractures in older adults, mainly in those at risk for or with established osteoporosis [8]. Likewise, an adequate intake of vitamin D and protein may decrease bone remodeling, thereby mitigating the risk of age-related bone fractures [9,14^▪▪^,^15^^▪▪^].

## EPIDEMIOLOGY AND DIAGNOSIS OF LACTOSE INTOLERANCE AND AGE-SPECIFIC TRENDS

Lactose is the main source of carbohydrates in human milk [16]. Digestion and absorption of lactose takes place after its cleavage into glucose and galactose by lactase expressed on the brush border of small bowel enterocytes, mainly in the mid-jejunum [1]. Lactase expression in the small intestine decreases physiologically during life in most populations [17,18].

Lactose malabsorption consists in a reduced absorption of lactose in the intestinal tract because of different causes, whereas lactose intolerance is characterized by the occurrence of gastrointestinal symptoms resulting from colonic fermentation of undigested lactose [1].

It has been estimated that lactose malabsorption occurs in around two-thirds of the world's population, with a wide variation in prevalence among countries and regions, ranging from 28% (19–37) in Western, Southern, and Northern Europe to 70% (57–83) in the Middle East [19]. However, these percentages may underestimate or overestimate the real prevalence, since the lack of a gold standard for diagnosis. In general, the frequency of lactose malabsorption increases with age, mainly in in populations with a high prevalence. Although the physiological and progressive decline of lactase activity in the intestinal brush border has been firstly implicated in this phenomenon [20], other causes have been suggested, such as the higher prevalence of small intestinal bacterial overgrowth (SIBO) in older adults, as later discussed [19].

The most common test for diagnosing lactose malabsorption is the hydrogen breath test (HBT). The rationale of this investigation is based on detection of hydrogen in pulmonary expired air produced from the fermentation of undigested lactose by the colonic microflora [1,18].

Other diagnostic tests, rarely used in clinical practice because of their invasiveness, low reliability. or low diagnostic accuracy, are the direct measurement of lactase enzyme activity in small intestinal tissue biopsies and the genetic test for the -13910 C/T polymorphism [21]. Both of them may assess a condition of adult-type hypolactasia; however, their results do not necessarily reflect the clinical picture [21].

## EFFECTS OF AGING ON LACTOSE DIGESTION AND ABSORPTION AND AGE-RELATED DISEASE MECHANISMS

Despite growing knowledge on lactose malabsorption and intolerance, correct approach to these conditions, including their diagnostic assessment, remains a matter of debate, especially in older adults.

Few studies have been conducted to establish possible age-related differences in lactose metabolism. However, no definitive conclusions or recommendations for clinical practice are available on the subject. Earlier studies found a significant greater incidence of positive lactose HBT in older individuals compared with younger adults [22,23]. Yet, intolerant individuals with severe gastrointestinal symptoms represented the minority [23].

As previously mentioned, both lactose malabsorption and intolerance may be influenced by several factors, some of which are more evident in older adults. For instance, a delayed small intestine transit time and the occurrence of SIBO, may interfere with H_2_ production capacity [23]. By excluding these unrelated factors responsible for lactose malabsorption, Di Stefano *et al.*[23] showed higher prevalence of lactose malabsorption in older adults. However, as already reported, also these authors found that the older adults experienced a lower prevalence of intolerance symptoms compared with younger individuals, with an overall limited symptom score in the malabsorber group [23].

The mechanisms underlying the higher prevalence of lactose malabsorption in old age are not completely clear. It has been suggested that this phenomenon may result from the ‘aging gut process’, characterized by elevated enterocyte turnover with consequent increased percentage of immature villous epithelial cells with reduced lactase expression [23].

## DIFFERENTIAL DIAGNOSIS OF LACTOSE INTOLERANCE AND THEIR RELATIONSHIP WITH AGING

Although HBT represents the most common diagnostic test for diagnosis of lactose malabsorption, it can also lead to false-positive results, linked to an increased H_2_ production due to a condition of small intestinal bacterial overgrowth [18,24], and false-negative results, for example, in no H_2_ producers. The small intestinal bacterial overgrowth may be responsible for the most of false-positive lactose HBT results, as it may share several clinical features with lactose intolerance [18]. The actual prevalence of SIBO in old and very old individuals is at present unknown. However, the main mechanisms underlying SIBO pathogenesis are typical of the aging process, including a high prevalence of motility alterations or anatomical changes of the intestine (i.e. after surgery), adherences, diverticular disease, and immunological deficits [25,26^▪▪^]. Diabetes mellitus, Parkinson's disease, hypothyroidism, and depression are common conditions associated with advancing age that affect the autonomic nervous system, slowing the gastrointestinal transit and consequently increasing the probability to develop a SIBO condition. As a matter of fact, about half of individuals with Parkinson's diseases has a diagnosis of SIBO [25,26^▪▪^].

In this regard, Almeida *et al.*[27] showed that, although the prevalence of lactose malabsorption evaluated by HBT was higher in 20 healthy old volunteers compared with a younger control group, also the prevalence of SIBO was higher in the older group and did not correlate with lactase levels in small bowel biopsy specimens.

Moreover, a positive HBT indicative of lactose malabsorption does not necessarily reflect lactose intolerance [28]. The clinical picture of lactose intolerance may be influenced by multiple factors, such as the amount of lactose in the intestinal lumen, gastrointestinal motility, composition on intestinal microflora, and visceral hypersensitivity [1].

Hence, a careful evaluation of gastrointestinal symptoms (i.e. abdominal pain, nausea, bloating, borborygmi, and diarrhea) in response to ingestion of a predefined amount of lactose is of utmost importance to limit the number of confounding and inconclusive diagnostic tests. Self-reported milk intolerance alone does not provide satisfactory results, with a sensitivity of 30–51% and specificity of 25–87% [29]. Several confounding factors may interfere with this evaluation, and many individuals may mistakenly attribute gastrointestinal symptoms to lactose intolerance. Far from simplifying such a complicated issue, an actual condition of lactose intolerance may be strongly suggested by a close temporal relationship between the ingestion of milk or dairy products and the onset of symptoms. Moreover, diarrhea, abdominal pain, bloating, urgency, and flatulence, may represent typical symptoms of radiation-induced bowel injury; however, they are often associated with bleeding and weight loss. In this case, the temporal relationship may help in differential diagnosis, as acute symptoms usually occur within 3 months from starting of radiotherapy and often resolve within a few months, although a small percentage of patients undergoing radiotherapy may experience chronic or late-onset gastrointestinal symptoms [30]. As the same time, drug-induced enteritis may manifest with diarrhea or other nonspecific clinical presentation, making this condition worthy to consider in differential diagnosis of lactose malabsorption, also in consideration of the increasingly widespread use of medications, mainly antibiotics [31].

The psychological features of patients should also be carefully assessed. Indeed, anxiety and depression can frequently contribute to amplifying a mild discomfort or lead to misinterpreting the eventual occurrence of symptoms related to lactose ingestion [29].

## CLINICAL IMPLICATIONS

Based on the above-mentioned considerations, it becomes clear that an accurate collection of the medical history represents the first step to guide the correct diagnostic workup of lactose malabsorption and intolerance.

Lactose HBT is a noninvasive and inexpensive diagnostic test widely available in clinics. However, its reliability may be negatively impacted by advanced age, as it requires an adequate patient compliance, of at least 4 h, and the need to move from home to a dedicated healthcare facility. Therefore, even more than for younger adults, the indication to a diagnostic test for lactose malabsorption requires to be carefully evaluated in older individuals. On the other hand, considering the known decrease of prevalence of intolerance symptoms with advancing age [23], a thorough clinical evaluation may reduce the need for diagnosing lactose malabsorption by HBT.

All these evaluations are of crucial importance to balance the effort in improving the patient clinical picture and dietary restrictions. In an earlier work, we proposed that adults with both a diagnosis of lactose malabsorption and confirmed intolerance symptoms may reduce or avoid ingesting milk and dairy products to obtain symptom remission but only for a limited period [32]. Then, a gradual re-introduction of dairy products should be recommended to assure an adequate intake of essential nutrients, such as calcium and vitamin D, if the remaining diet is not sufficient to compensate for the restriction. Because of the detrimental impact of inadequate dietary intake in older adults, concerns about a lactose-free diet becomes more relevant in this population. An adequate counselling is mandatory in such a context where improper lifestyle habits, social isolation, and related psychological factor may contribute to reducing food consumption, with a significant impact on health [29,32,33^▪▪^,^34^]. The overall mean protein, calcium, vitamin D, and vitamin B12 intakes are usually quite low in older adults, but in particular among nondairy users [34].

Appropriate advices should be provided to assess the individual threshold dose and to facilitate its gradual rise, such as ingestion of milk together with other foods, the distribution of small daily milk amount across meals, the addition of exogenous lactase to milk, or the consumption of yogurt that normally contains lactase, or hard cheeses and other fermented dairy products [32,35].

In fact, small and more physiological doses (12–15 g/day, about 240 ml of milk) of the sugar usually did not result in a significant increase in the severity of any symptom over that noted at baseline [1,16]. Finally, regular consumption of lactose in malabsorbers could act as a prebiotic, promoting the growth of beneficial commensal bacteria, such as *Bifidobacteria* and *Lactobacillus* accompanied by a proportional reduction of the genus *Bacteroides* and *Clostridia,* thus contributing to colonic microflora balance [36].

Based on these considerations, testing for lactose malabsorption in individuals already avoiding dairy products for a suspected lactose intolerance hardly influences their dietary habits. Rather, psychological, cultural, and behavioral factors should be promptly assessed to support these patients, mainly if old, in adopting adequate dietary pattern.

## CONCLUSION

The management of lactose malabsorption and intolerance in older adults deserves a personalized approach.

Because of the importance of maintaining an adequate nutritional status in this population, efforts should be made to avoid overly restrictive diets. Like in younger adults, the first step in approaching older patients complaining of symptoms suggestive of lactose intolerance, is represented by an accurate collection of medical history. The presence of confounding factors, such as predisposing conditions for SIBO, concomitant diseases or drugs affecting intestinal motility, physiological factors, should be preliminary evaluated.

Obtaining a lactose HBT may be unnecessary in most cases of suspected lactose intolerance, also considering the low reliability of the test in older adults and the high rate of false-positive results. In general, the main recommendation is to limit dietary restriction to selected cases. Awareness of the importance of an adequate nutrition for older adults should be implemented by advising the adoption of healthy, nutrient-rich diets and correct lifestyle.

## Acknowledgements

*None.*

### Financial support and sponsorship

*None.*

### Conflicts of interest

*There are no conflicts of interest.*

## REFERENCES AND RECOMMENDED READING

Papers of particular interest, published within the annual period of review, have been highlighted as:

▪ of special interest
▪▪ of outstanding interest
